# Longitudinal evaluation of whole blood miRNA expression in firefighters

**DOI:** 10.1038/s41370-021-00306-8

**Published:** 2021-02-18

**Authors:** Alesia M. Jung, Jin Zhou, Shawn C. Beitel, Sally R. Littau, John J. Gulotta, Darin D. Wallentine, Paul K. Moore, Jefferey L. Burgess

**Affiliations:** 1 Department of Epidemiology and Biostatistics, Mel and Enid Zuckerman College of Public Health, University of Arizona, Tucson, AZ USA; 2 Department of Community, Environment and Policy, Mel and Enid Zuckerman College of Public Health, University of Arizona, Tucson, AZ USA; 3 Tucson Fire Department, Tucson, AZ USA

**Keywords:** Cancer, Epidemiology, Workplace Exposures

## Abstract

**Background:**

Dysregulated microRNA (miRNA) expression could provide a mechanism linking firefighter exposure to increased cancer risk.

**Objective:**

To determine if changes in longitudinal miRNA expression in firefighters are associated with occupational exposures.

**Methods:**

Whole blood MiRNA was evaluated in 52 new recruits prior to live-fire training and 20–37 months later. Linear mixed effects models adjusted for age, ethnicity, BMI, and batch effects were used to determine associations separately for all fires and structure fires only between employment duration, cumulative fire-hours and fire-runs, and time since most recent fire with (1) nine a priori and (2) the full array of 799 miRNAs.

**Results:**

For multivariable models including all fires, two a priori miRNAs were associated with employment duration and four with time since most recent fire. For multivariable models restricted to structure fires, three a priori miRNAs were associated with employment duration and one with fire-runs. Additional miRNAs from the full array were associated with employment duration for all fires and/or structure fires. In general, tumor suppressive miRNAs decreased and oncogenic miRNAs increased with exposure.

**Significance:**

Changes in miRNAs may serve as biomarkers of exposure effects and a mechanism for increased cancer risk in firefighters.

## Introduction

Firefighting is a hazardous occupation involving exposure to toxic combustion byproducts, including many known and probable carcinogens [1–5]. Within the past decade, the epidemiological evidence that cancer risk is elevated among firefighters compared to the general population has grown [6–10]. Notably, an evaluation of ~30,000 career firefighters in the United States (US) found excess overall cancer mortality and incidence (increases of 9 and 14%, respectively), in addition to increased incidence and mortality for cancers of the lung, digestive tract, and kidney [6]. Associations between lung cancer and leukemia mortalities and surrogate fireground exposures were also reported, specifically cumulative fire-hours and cumulative fire-runs, respectively [7]. A meta-analysis study reported elevated risk in firefighters of cancers of the prostate, testes, colon, rectum, bladder, thyroid, and pleura as well as non-Hodgkin lymphoma and melanoma [11]. Other studies using cancer registry data from California and Florida have also observed elevated cancer risks among firefighters, including melanoma, multiple myeloma, leukemia, and esophageal, prostate, brain, and kidney, testicular, thyroid and colon cancers [9, 10].

Despite the number of epidemiologic studies linking firefighting to excess cancer risk, there is limited information on the cellular mechanisms that lead to cancer in firefighters. Partly due to this lack of mechanistic evidence, an assessment by the International Agency for Research on Cancer concluded that firefighting was only possibly carcinogenic in humans [12]. Epigenetic modifications, such as dysregulated microRNA (miRNA) expression and DNA methylation, are changes in gene expression that are heritable, reversible (do not affect DNA sequence), and critical steps in the cancer pathway that have been associated with the regulation of oncogenes and tumor suppressor genes [13–15]. These epigenetic modifications have been proposed as biomarkers of cancer risk and exposure, and a limited number of studies have previously examined epigenetic modifications among firefighters [16–18].

MiRNAs are small non-coding RNAs involved in the regulation of cell cycle progression, apoptosis, and cell differentiation, with reported oncogenic or tumor-suppressive roles, though these may vary depending on which gene is targeted and which cancer is considered [19–21]. Our previous research comparing incumbent and new recruit firefighters identified nine dysregulated miRNAs with previously published associations with cancer or cancer pathways [16]. However, there are no prior published studies evaluating potential associations between longitudinal changes in miRNA and interim firefighting exposures. This information could be useful to advance future efforts to identify firefighters at increased cancer risk, to provide intermediate endpoints for cancer prevention interventions, and to provide evidence for causation in firefighter workers’ compensation cancer claims.

In our current analysis we evaluated whole blood samples, collected from newly recruited firefighters prior to live-fire training and again ~2 years later, for miRNA expression levels and determined if changes in expression were associated with measures of occupational exposure. We hypothesized that after ~2 years of employment in the fire service, we would be able to identify differential miRNA expression patterns among newly recruited firefighters that were associated with employment duration, surrogate measures of fire exposure (cumulative hours at fire and cumulative number of fires), and/or most recent fire exposure, and that changes in these miRNAs would be associated with increased cancer risk based on published studies in the general population.

## Materials and methods

### Study participants

All study protocols and materials were approved by the University of Arizona Institutional Review Board (IRB). Study participants provided written informed consent. New-recruit firefighters with no previous live-fire exposures were recruited from the Tucson Fire Department (TFD) between 2015 and 2016. Participant age, race, ethnicity, height, and weight were collected from health records and current tobacco usage was assessed at study enrollment via survey. Participants who reported current tobacco usage were excluded from analyses. Height and weight were used to calculated body mass index (BMI) (kg/m^2^) and classified using World Health Organization categories: normal (18.5–24.9), overweight (25.0–29.9) and obese (≥30). Biological samples including blood, urine and buccal cells were collected at baseline and blood samples were collected again after a period of 20 to 37 months, at which time a follow-up survey was administered. Employment duration, the total months between baseline and follow-up, was calculated.

### Measures of fireground exposures

Cumulative hours at fire (fire-hours) and cumulative number of fires (fire-runs) were collected from TFD response records and assessed as surrogate measures of chronic fireground exposure. This information included the type of fire (structure fire, vehicle fire, other), date and time of the fire, and duration of the fire response (minutes). The time between the blood draw at follow-up and the new recruit’s most recent response to a fire call (days since most recent fire exposure) was calculated. Fire-hours, fire-runs, and days since most recent fire exposure were determined for: (1) all fire types (structure, vehicle, and other); (2) structure fires only; and (3) non-structure fires only. Other fires, or non-structure non-vehicle fires, consisted of outside vegetation (i.e., grass, brush, forest, crop) fires, outside trash fires, outside fires involving property of value (e.g., storage, equipment), and unclassified fires.

### MicroRNA expression measurement

The protocols used for sample collection and processing have been previously reported [16]. Levels of miRNA expression were measured using an nCounter Human v3 miRNA expression panel (NanoString Technology, Inc., Seattle, WA), a profile of 799 curated and clinically relevant human miRNAs from miRBase v21, in addition to 5 housekeeping genes and 20 assay controls (6 positive, 8 negative, and 6 ligation controls) [22, 23] in four batches analyzed in 2016, 2017, 2018, and 2019. The expression panel accounts for more than 95% of all observed sequencing reads in miRBase 21 [24]. Raw counts from each gene were normalized against background genes. The overall assay performance was assessed through evaluation of positive controls.

### Statistical analysis

In order to normalize and remove unwanted variation (batch effects), we adopted the Removing Unwanted Variation-III method (RUV-III), which makes vital use of pseudo-replicates and control genes [25]. In our miRNA design, we purposely measured the same sample at all four time points (2016, 2017, 2018 and 2019), serving as pseudo-replicates. The NanoString platform also includes housekeeping genes and negative and positive control genes on their array. RUV-III (i) takes residuals from the replicate expression measurements and estimates one aspect of the unwanted variation; (ii) takes the results of (i) together with the expression values of the negative controls, and estimates another aspect of the unwanted variation; and (iii) combines the results from (i) and (ii) into an estimate of the unwanted variation and subtracts that from the data. Relative Log Expression (RLE) plots were used to diagnosis batch correction, using the “*ruv*” R package (https://cran.r-project.org/package=ruv). RUV-III corrected miRNAs were then analyzed using the limma software package [26] to determine the association between fireground exposures and miRNA expression at follow-up to expression at baseline. Linear mixed effects models with Empirical Bayes estimators were adopted and adjusted for age, ethnicity, BMI, and batch effects [27]. MiRNAs were considered to be differentially expressed if the *p* value was less than 0.05 after Bonferroni adjustment [28]. All statistical analyses were performed using R (version 3.4.1).

The exposures of interest were employment duration, measures of chronic fireground exposure (fire-hours and fire-runs) and acute fireground exposure (days since most recent fire exposure). Employment duration was included as a proxy for cumulative firefighter exposures of all types, including but not limited to chemical exposures and shift-work. Fire-runs and fire-hours were considered the fireground exposures of greatest interest, due to their use as measures of cumulative fireground exposure in previous firefighter research [7, 29]. These exposures were treated as continuous variables in our models. Days since most recent fire was included in our models to adjust for potential confounding of acute exposures on the association between chronic fireground exposures and differential miRNA expression. As a novel potential confounder, several definitions were considered for days since most recent fire: continuous log-transformed days, categorized at the median value, categorized at tertile values, and categorized at quartile values. For categorical definitions, the categories representing the longest number of days since fire exposure (more than the median, the third tertile, the fourth quantile) served as the reference value. The definition that explained the most variation of data for each miRNA was selected based on highest Akaike information criteria (AIC), using separate models adjusted for age, BMI and ethnicity [30].

We also hypothesized that firefighters would face increased risk of exposure from structure fires compared to vehicle or other fire types. Previous research has shown that median urinary concentrations of polycyclic aromatic hydrocarbon (PAH) metabolites after a fire incident were greatest among firefighters who performed interior operations at structural fires, such as fire attack and rescue [1] and that mean respirable particles measured at vehicle fires was lower than at structural fires [2, 3]. Therefore, we stratified analyses by fire type (all fires, or structure fires only). For analyses considering all fires, all fire-hours, all fire-runs, and days since most recent fire were included as covariates, in addition to age, BMI and ethnicity. For analyses considering only structure fires, structure fire-hours, structure fire-runs, and days since most recent structure fire were included as covariates, in addition to age, BMI and ethnicity.

The outcome of interest was differential expression of miRNAs. Two sets of miRNA markers were considered: (1) nine a priori markers significant in our previous analysis comparing new recruits to incumbent firefighters [16]; and (2) the full array of miRNAs. The effects of employment duration, fire-hours, fire-runs, and days since most recent fire exposure on differential miRNA expression were first evaluated in separate models adjusted for age, ethnicity, BMI, batch effects and Bonferroni correction (referred to as the univariable models) to compare to values reported in our previous cross-sectional study of incumbent and new recruit firefighters [16]. Second, models containing employment duration, either fire-hours or fire-runs, and days since most recent fire, in addition to age, ethnicity, BMI, batch effects and Bonferroni correction (referred to as the multivariable models) were run. Fire-hours and fire-runs were expected to be highly correlated, so they were examined in separate multivariable models. Differential expression, comparing expression at follow-up to expression at baseline, was presented as log-fold changes (log_2_FCs) and fold changes (FCs) with accompanying *p* values and 95% confidence intervals (95% CIs). Based on Bonferroni correction, *p* values < 0.05/n (where n is the number of statistical tests performed) were considered statistically significant.

Our previous study presented select cancer associations for the nine a priori miRNAs [16]. To interpret findings from our current analyses of the full array of miRNAs, the select cancer associations of significant full array miRNAs with an absolute FC >1.25 were also evaluated. Given that previous studies have used absolute FC >1.5 to identify potential diagnostic and prognostic miRNAs in cancer samples [31–33], we assumed that 25% might represent a reasonable threshold for increased risk of exposure. When published evidence of an association between miRNA expression and cancer risk could be found, one reference per miRNA was presented. The reference was selected regardless of reported direction of association (increased or decreased FC), instead based on the following additive criteria: (1) the study utilized blood samples, (2) the association was also validated in an independent sample set, (3) the association with the specific cancer was also found in other studies, and (4) the association with the specific cancer was also found using other sample types (e.g., tissue and/or cell lines). In the case that no published studies using blood samples could be found, the criteria were applied to studies using tissue samples.

## Results

### Study participants and occupational exposures

Of 90 new recruit firefighters offered participation, 89 (99%) consented and were enrolled. Twenty (22%) of the 89 were excluded because they lacked a blood sample collected at baseline or follow-up, 10 (11%) were excluded because they left the fire department before follow-up, 3 (3%) were excluded due to poor RNA yield, and 4 (4%) were excluded because they failed quality control testing for miRNA, leaving 52 individuals (58%) in the study. The participants were all male, mostly white and non-Hispanic (Table 1). At study enrollment, the average participant was 28.2 years of age. The period of follow-up ranged from 20 to 37 months. For all fire types, the average time spent at all fires combined was 27.0 h and the average number of fires was 49.2. The median time between the follow-up blood sampling and most recent fire was 26 days. Employment duration was moderately correlated with time at fires, fire-runs, and days since most recent fire, with correlation coefficients of 0.43, 0.46, and 0.36, respectively. Time at fires was highly correlated with fire runs but not days since most recent fire, with correlation coefficients of 0.86 and 0.21, respectively. Fire runs and days since most recent fire had low correlation (coefficient 0.06).

**Table 1:** Characteristics of new recruit firefighters at enrollment, *N* = 52.

	*N* (%)
Age (years)	
≤29	37 [71]
30–39	11 (21)
≥40	4 (7)
Mean ± SD (range: min–max)	28.2 ± 6.0 (19.1–45.6)
Body mass index (kg/m^2^)	
Normal (18.5–24.9)	18 (15)
Overweight (25.0–29.9)	28 [54]
Obese (≥30)	6 (11)
Mean ± SD	26.4 ± 3.7
Race	
White	50 [96]
Other	2 (7)
Ethnicity	
Hispanic	5 (10)
Non-Hispanic	47 [90]
Fire exposure between enrollment and follow-up	
Average employment duration (months) ± SD	26.5 ± 4.3
All fire incident types	
Average cumulative time at fire (hours) ± SD^a^	27.0 ± 8.6
Average cumulative number of fire runs ± SD^a^	49.2 ± 15.0
Days since most recent fire at follow-up, median (IQR)	26 (15.8, 112.5)
Structure fire incidents only	
Average cumulative time at structure fires (hours) ± SD^a^	13.2 ± 6.7
Average cumulative number of structure fire runs ± SD^a^	15.7 ± 7.3
Days since most recent structure fire, median (IQR)	41.5 (18, 141.5)

*SD* standard deviation, *IQR* interquartile range.

^a^Length of time over which this value accumulated varied by individual (range: 20–37 months).

When restricted to structure fires, the average time spent at all structure fires combined over the follow-up period was 13.2 h, the average number of structure fire-runs was 15.7, and the median time since most recent structure fire was 41.5 days. Employment duration was not highly correlated with time spent at structure fires, structure fire-runs, and days since most recent structure fire (correlations coefficients of 0.19, 0.23, and 0.04, respectively). However, time spent at structure fires was highly correlated with structure fire-runs (correlation coefficient 0.89) as expected, while days since most recent structure fire had low correlation with either structure fire-hours or structure fire-runs (correlation coefficients 0.14 and 0.01, respectively).

#### Fireground exposure and differential miRNA expression

Results are provided separately for all fires and structure fires only. The univariable and multivariable model results restricted to non-structure fires closely followed results of the all fire models, with the same significant miRNAs for each of the models, and the same direction of the coefficients.

#### Univariable analyses of a priori markers, all fires

**Fig. 1:**
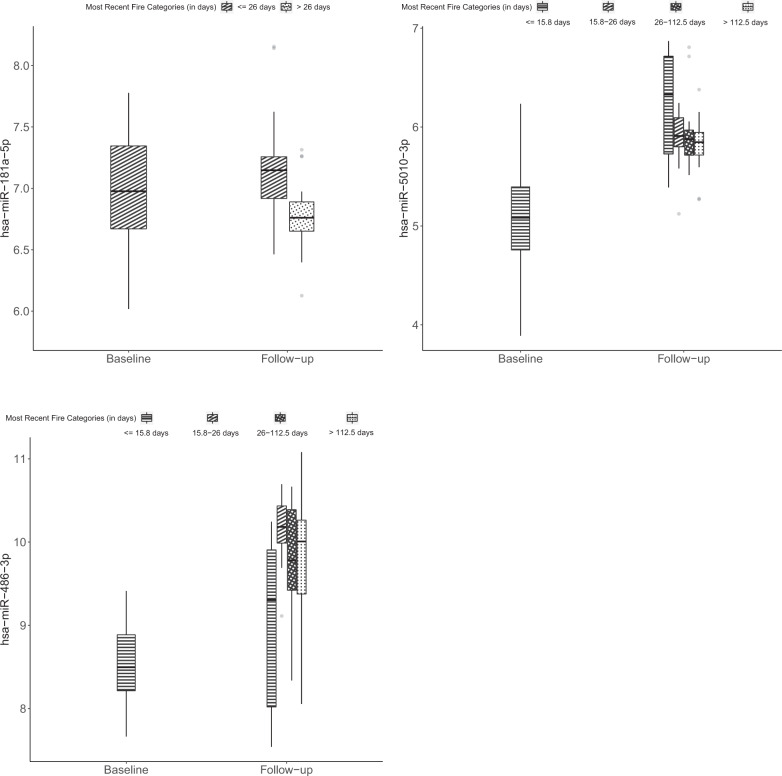
Expression of microRNAs (miRNAs) among firefighters with fold-changes significantly associated with days between most recent fire and follow-up blood draw. MiRNA expression of miR-181a-5p, miR-5010-3p, and miR-486-3p was measured among 52 new recruit firefighters prior to live-fire training and again ~2 years later using an nCounter Human v3 miRNA expression panel. Within boxplots, the center horizontal line represents the median value and dots represent outliers.

In our univariable models (adjusted for age, ethnicity, BMI, batch effects, and multiple comparisons) considering all fire incidents, employment duration, fire-hours, and fire-runs were all significantly associated with differential expression of the same four a priori miRNAs (Table 2). These included one miRNA with decreased expression (miR-145-5p) and three with increased expression (miR-548h-5p, miR-5010-3p, and miR-486-3p). Days since most recent fire exposure was associated with three of the nine a priori miRNAs (miR-181a-5p, miR-5010-3p, and miR-486-3p). Levels of these three miRNAs comparing baseline and days since most recent fire categories are shown in Fig. 1.

**Table 2:** Differential miRNA expression of a priori miRNAs by fire type exposure: separate models adjusted for age, BMI, ethnicity, batch effects.

miRNA	Incumbents vs new recruits^a^			New recruits (follow-up vs baseline measurement)^b^											
				Employment duration			Fire-hours			Fire-runs			Most recent fire^c^		
All fires	FC	95% CI		FC	95% CI		FC	95% CI		FC	95% CI		FC	95% CI	
hsa-miR-1260a	**0.55**	**0.43**	**0.71**	0.96	0.93	1.00	0.98	0.94	1.01	0.95	0.89	1.01	0.86	0.69	1.07
hsa-miR-548h-5p	**0.59**	**0.51**	**0.69**	**1.05**	**1.02**	**1.08**	**1.04**	**1.02**	**1.07**	**1.09**	**1.04**	**1.14**	1.02	0.83	1.26
hsa-miR-145-5p	**0.44**	**0.32**	**0.61**	**0.96**	**0.93**	**0.98**	**0.97**	**0.94**	**0.99**	**0.94**	**0.90**	**0.98**	0.83	0.72	0.96
hsa-miR-4516	**0.56**	**0.48**	**0.65**	0.97	0.95	0.99	0.98	0.96	1.00	0.96	0.93	1.00	0.92	0.82	1.03
hsa-miR-331-3p	**0.60**	**0.52**	**0.70**	0.97	0.94	0.99	0.98	0.96	1.00	0.96	0.92	1.00	0.84	0.70	1.01
hsa-miR-181a-5p	**0.62**	**0.53**	**0.72**	0.99	0.97	1.02	0.99	0.97	1.02	0.99	0.95	1.03	**0.81**	**0.70**	**0.92**
has-miR-5010-3p	**1.59**	**1.41**	**1.81**	**1.13**	**1.10**	**1.16**	**1.10**	**1.08**	**1.31**	**1.20**	**1.15**	**1.26**	**1.44**	**1.14**	**1.82**
hsa-miR-374a-5p	**1.72**	**1.40**	**2.13**	1.03	0.99	1.07	1.03	1.00	1.06	1.05	0.99	1.11	1.09	0.89	1.34
hsa-miR-486-3p	**3.35**	**2.59**	**4.33**	**1.15**	**1.10**	**1.21**	**1.12**	**1.07**	**1.16**	**1.23**	**1.14**	**1.32**	**2.20**	**1.66**	**2.91**
**Structure fires**															
hsa-miR-1260a	**0.55**	**0.43**	**0.71**	0.96	0.93	1.00	0.94	0.83	1.05	0.94	0.85	1.04	1.00	0.81	1.25
hsa-miR-548h-5p	**0.59**	**0.51**	**0.69**	**1.05**	**1.02**	**1.08**	**1.17**	**1.08**	**1.28**	**1.13**	**1.05**	**1.22**	1.12	0.94	1.35
hsa-miR-145-5p	**0.44**	**0.32**	**0.61**	**0.96**	**0.93**	**0.98**	0.93	0.86	1.01	0.94	0.88	1.00	0.82	0.66	1.00
hsa-miR-4516	**0.56**	**0.48**	**0.65**	0.97	0.95	0.99	0.94	0.89	1.00	0.95	0.90	1.00	0.90	0.77	1.06
hsa-miR-331-3p	**0.60**	**0.52**	**0.70**	0.97	0.94	0.99	0.94	0.87	1.01	0.95	0.89	1.01	**0.83**	**0.70**	**0.99**
hsa-miR-181a-5p	**0.62**	**0.53**	**0.72**	0.99	0.97	1.02	1.01	0.94	1.09	1.00	0.94	1.07	**0.74**	**0.61**	**0.90**
has-miR-5010-3p	**1.59**	**1.41**	**1.81**	**1.13**	**1.10**	**1.16**	**1.35**	**1.24**	**1.48**	**1.30**	**1.20**	**1.39**	1.37	1.07	1.77
hsa-miR-374a-5p	**1.72**	**1.40**	**2.13**	1.03	0.99	1.07	1.07	0.97	1.20	1.07	0.97	1.16	1.10	0.89	1.35
hsa-miR-486-3p	**3.35**	**2.59**	**4.33**	**1.15**	**1.10**	**1.21**	**1.38**	**1.21**	**1.60**	**1.35**	**1.20**	**1.52**	**1.81**	**1.33**	**2.45**

Associations significant after Bonferroni correction are in bolded text.

*FC* fold-change, *CI* confidence interval.

^a^A priori markers were identified and originally presented in Jeong et al.[16].

^b^Models also adjusted for age, BMI, ethnicity, batch effects, and Bonferroni correction. For employment duration, effect is for a 6-month increase. For fire-hours, effect is for a 10-h increase. For fire-runs, effect is for a 10-fire increase.

^c^The best measure of time since most recent fire exposure (continuous log-days, split at median, split at tertiles, or split at quantiles) was selected for each miRNA by the highest Akaike information criteria (AIC) value. For time since most recent fire exposure, effect is the earliest exposure compared to later exposure(s). hsa-miR-548h-5p and hsa-miR-374a-5p used time split at the median. Hsa-miR-1260a, hsa-miR-145-5p, hsa-miR-4516, hsa-miR-331-3p, hsa-miR-181a-5p, hsa-miR-5010-5p, and hsa-miR-486-3p used time split at quantiles.

#### Univariable analyses of a priori markers, structure fires only

In univariable models (adjusted for age, ethnicity, BMI, batch effects, and multiple comparisons) considering only structure fire incidents, structure fire-hours and structure fire-runs were associated with differential expression of three out of four of the same a priori miRNAs from the analysis of all fire incidents, with miR-145-5p losing statistical significance (Table 2). Days since most recent structure fire were associated with three a priori miRNAs.

#### Multivariable analyses of a priori markers, all fires

Many of the associations seen in our univariable a priori models lost significance in the multivariable models (Table 3). Employment duration was only significantly associated with miR-5010-3p in the fire-hours and fire-runs models, after adjustment for days since most recent fire exposure, age, BMI, and ethnicity. None of the nine a priori miRNAs were significantly associated with fire-hours or fire-runs after adjustment for Employment duration, days since most recent fire exposure, and age, BMI, and ethnicity. Days since most recent fire exposure was associated with miR-181a-5p and miR-486-3p in the fire-hours and fire-runs models. R^2^ values for the models varied from 0.056 to 0.472.

**Table 3:** Multivariable parameter estimates of differential miRNA expression for a priori markers: employment duration, fire exposures and most recent fire exposure^a,b^.

miRNA	Model with fire-hours							Model with fire-runs						
	Employment duration		Fire-hours		Most recent fire		*R*^2^	Employment duration		Fire-runs		Most recent fire		*R*^2^
	β	*p* value	β	*p* value	β	*p* value		β	*p* value	β	*p* value	β	*p* value	
**All fires**														
hsa-miR-1260a	−0.082	0.342	0.055	0.673	−0.061	0.754	0.056	−0.137	0.117	0.085	0.268	−0.096	0.624	0.064
hsa-miR-548h-5p	−0.009	0.892	0.166	0.097	−0.266	0.130	0.182	0.051	0.443	0.037	0.525	−0.287	0.110	0.162
hsa-miR-145-5p	−0.076	0.179	0.041	0.633	−0.105	0.416	0.115	−0.055	0.340	0.003	0.947	−0.103	0.433	0.112
hsa-miR-4516	−0.045	0.312	0.008	0.902	0.014	0.892	0.072	−0.046	0.308	0.006	0.880	0.0116	0.909	0.072
hsa-miR-331-3p	−0.059	0.298	0.088	0.301	−0.210	0.201	0.152	−0.085	0.136	0.076	0.124	−0.235	0.145	0.163
hsa-miR-181a-5p	−0.022	0.673	0.085	0.284	−**0.398**	**0.001**	0.146	−0.005	0.921	0.032	0.483	−**0.406**	**0.001**	0.140
hsa-miR-5010-3p	**0.182**	**0.003**	0.007	0.935	−0.121	0.491	0.449	**0.212**	<**0.001**	−0.025	0.633	−0.115	0.510	0.450
hsa-miR-374a-5p	0.039	0.632	0.011	0.931	−0.011	0.953	0.046	−0.008	0.926	0.050	0.484	−0.034	0.855	0.049
hsa-miR-486-3p	0.080	0.360	−0.077	0.559	**1.015**	<**0.001**	0.463	0.145	0.098	−0.109	0.155	**1.048**	<**0.001**	0.472
**Structure fires**														
hsa-miR-1260a	−0.178	0.006	0.272	0.122	0.262	0.179	0.108	−**0.207**	**0.004**	0.299	0.078	0.271	0.163	0.113
hsa-miR-548h-5p	0.065	0.177	0.099	0.459	−0.154	0.304	0.181	0.100	0.071	−0.005	0.967	−0.131	0.380	0.174
hsa-miR-145-5p	−0.112	0.010	0.147	0.223	0.043	0.749	0.132	−0.125	0.012	0.159	0.172	0.049	0.713	0.134
hsa-miR-4516	−0.053	0.125	0.015	0.878	0.089	0.403	0.079	−0.061	0.113	0.035	0.701	0.088	0.403	0.080
hsa-miR-331-3p	−0.111	0.008	0.204	0.075	−0.059	0.641	0.140	−0.117	0.014	0.241	0.037	−0.195	0.202	0.158
hsa-miR-181a-5p	−0.020	0.640	0.244	0.027	−**0.666**	<**0.001**	0.188	−0.013	0.776	0.189	0.081	−**0.656**	<**0.001**	0.174
hsa-miR-5010-3p	**0.230**	<**0.001**	−0.052	0.659	−0.261	0.051	0.472	**0.247**	<**0.001**	−0.088	0.440	−0.248	0.061	0.473
hsa-miR-374a-5p	−0.033	0.581	0.192	0.250	0.014	0.941	0.088	−0.051	0.453	0.302	0.075	−0.114	0.610	0.112
hsa-miR-486-3p	**0.197**	**0.005**	−0.392	0.028	0.634	0.020	0.477	**0.254**	<**0.001**	−**0.504**	**0.004**	0.679	0.012	0.496

Models also adjusted for age, BMI, ethnicity, batch effects, and Bonferroni correction. Associations significant after Bonferroni correction are in bolded text.

A priori markers were originally identified in Jeong et al. [16].

^a^For employment duration, effect is for a 6 month increase. For fire-h, effect is for a 10 h increase. For fire-runs, effect is for a 10 fire increase.

^b^Based on the highest Akaike information criteria (AIC), the best measure of time since most recent fire was selected (continuous, split at median value, split at tertile values, or split at quartile values). Hsa-miR-1260a, hsa-miR-145-5p, hsa-miR-4516 and hsa-miR-5010-3p used time split at median value. Hsa-miR-548h-5p used time split at tertiles. Hsa-miR-331-3p, hsa-miR-181a-5p, hsa-miR-374a-5p and hsa-miR-486-3p used time split at quartiles.

#### Multivariable analyses of a priori markers, structure fires only

Most of the associations seen in the univariable analyses restricted to structural fires lost significance in the multivariable models (Table 3). Employment duration in both structure fire-hours and structure fire-runs models was significantly associated with changes in two a priori miRNAs (miR-5010-3p and miR-486-3p). Additionally, employment duration in models adjusted for structure fire-runs (but not structure fire-hours) was found to be associated with expression of miR-1260a. Increasing employment duration was associated with increased expression of miR-5010-3p and miR-486-3p and decreased expression of miR-1260a. In contrast to analyses of all fires, structure fire-runs, but not structure fire-hours was significantly inversely associated with differential expression of miR-486-3p after adjustment for employment duration and time since most recent structure fire. Time since most recent structure fire was significantly associated with miR-181a-5p. *R*^2^ values varied from 0.056 to 0.496.

#### Full miRNA array analyses

Restricted to structural fires, nine miRNAs from the full array with absolute fold changes >1.25 were associated with employment duration in multivariable analyses following full adjustment for either fire hours or fire runs, and days since most recent fire exposure, as well as age, BMI, and ethnicity (Table 4). These included eight miRNAs after adjusting for structure fire-hours and most recent structure fire and eight miRNAs after adjusting for structure fire-runs and most recent structure fire, with seven miRNAs in common. The expression of five miRNAs decreased and expression of four miRNAs increased longitudinally. Four of the five miRNAs with decreased expression have tumor suppressive roles in cancer and all miRNAs with increased expression have oncogenic roles in cancer. MiR-422a had decreased expression at follow-up and is reported to have a tumor suppressive role in colorectal cancer [34]. MiRNAs with increased expression (miR-525-3p, miR-548ad-3p and miR-548k) have reported oncogenic roles in hepatocellular, breast, and esophageal cancers, respectively [35–37]. Table 5 displays the log_2_FCs and *p* values for employment duration, time since most recent structure fire, and either fire-hours or fire-runs with miRNAs from the full array that were found to have be significantly associated with employment duration above the absolute FC of 1.25. We did not observe any significant associations between structure fire-hours or structure fire-runs and differential expression of miRNA, but changes in four miRNAs (three in the structure fire-hours models and three in the structure fire-runs models, with two overlapping) were associated with most recent structure fire exposure. In these adjusted models, *R*^2^ values varied from 0.480 to 0.795.

**Table 4:** Significant fold-changes of differential miRNA expression for full array markers: employment duration adjusted for chronic fireground exposure (structure fire-hours or structure fire-runs) and time since most recent structure fire^a,b,c^.

miRNA	Employment duration			Employment duration			Select cancer association^c^	Proposed role	Reference
	Adjusted for structure fire-hours and most recent structure fire			Adjusted for structure fire-runs and most recent structure fire					
	FC	95% CI		FC	95% CI				
hsa-miR-494-3p	0.60	0.54	0.66	0.65	0.59	0.72	Prostate*	Oncogene	Cai and Peng [39]
hsa-miR-422a	0.74	0.70	0.78	0.77	0.73	0.82	CRC*	Tumor suppressor	Zheng et al. [34]
hsa-miR-26a-5p	0.76	0.70	0.83	0.76	0.69	0.83	HCC*	Tumor suppressor	Tan et al. [53]
hsa-miR-92a-3p	0.78	0.73	0.83	0.79	0.73	0.84	CRC	Tumor suppressor	Slattery et al. [40]
hsa-let-7f-5p	–	–	–	0.80	0.73	0.87	CRC*	Tumor suppressor	Ghanbari et al. [54]
hsa-miR-548a-3p	1.30	1.16	1.46	–	–	–	Prostate*	Oncogene	Nguyen et al. [55]
hsa-miR-556-3p	1.32	1.17	1.48	1.34	1.18	1.53	Osteosarcoma*	Oncogene	Xie et al. [41]
hsa-miR-548ad-3p	1.38	1.21	1.58	1.39	1.20	1.60	Breast	Oncogene	Sugita et al. [36]
hsa-miR-525-3p	1.43	1.29	1.60	1.44	1.28	1.63	HCC	Oncogene	Augello et al. [35]

*FC* fold-change, *CI* confidence interval, *CRC* colorectal cancer, *HCC* hepatocellular carcinoma.

^a^Effect for employment duration is an increase of 6 months. Models adjusted for employment duration, chronic fire exposure (fire-hours or fire-runs), time since most recent fire (see footnote a), age, BMI, ethnicity, Bonferroni correction, and batch effects.

^b^An absolute FC >1.25 was applied to statistically significant miRNAs associated with length of service presented here.

^c^Cancer association shown was selected based on the following criteria: (1) when possible reported association based on serum samples rather than tissue samples or cell assays, (2) reported association was validated in at least one other dataset, and (3) association reported in multiple types of samples (serum, tissue, cell lines, etc). Select cancer associations based on circulating samples are indicated with an asterisk (*).

**Table 5:** Parameter estimates of statistically significant miRNAs from full miRNA expression panel associated with employment duration and adjusted for structure fire-hours or structure fire-runs and time since most recent structure fire^a,b,c^.

miRNA	Model with structure fire-hours							Model with structure fire-runs						
	Employment duration		Structure fire-hours		Most recent structure fire		*R*^2^	Employment duration		Structure-fire-runs		Most recent structure fire		*R*^2^
	β	*p* value	β	*p* value	β	*p* value		β	*p* value	β	*p* value	β	*p* value	
hsa-miR-494-3p	−0.735	<0.001	−0.392	0.025	−2.229	<0.001	0.778	−0.615	<0.001	−0.504	0.003	−2.247	<0.001	0.794
hsa-miR-422a	−0.439	<0.001	0.297	0.071	−1.321	<0.001	0.795	−0.370	<0.001	0.349	0.003	−1.346	<0.001	0.774
hsa-miR-26a-5p	−0.394	<0.001	0.272	0.120	0.403	<0.001	0.535	−0.403	<0.001	0.263	0.009	0.405	<0.001	0.756
hsa-miR-92a-3p	−0.366	<0.001	0.259	0.005	1.295	<0.001	0.618	−0.345	<0.001	−0.312	0.247	1.329	<0.001	0.611
hsa-let-7f-5p	–	–	–	–	–	–	–	−0.324	<0.001	0.243	0.007	0.388	0.008	0.443
hsa-miR-548a-3p	0.382	<0.001	0.259	0.103	0.398	0.017	0.480	–	–	–	–	–	–	–
hsa-miR-556-3p	0.396	<0.001	0.283	0.022	0.408	<0.001	0.407	0.424	<0.001	0.299	0.077	0.548	<0.001	0.408
hsa-miR-548ad-3p	0.469	<0.001	0.314	0.009	−1.960	0.008	0.426	0.471	<0.001	−0.403	0.002	−1.948	0.009	0.426
hsa-miR-525-3p	0.519	<0.001	−0.325	0.136	0.552	<0.001	0.568	0.529	<0.001	−0.338	0.118	−0.623	<0.001	0.570

Models also adjusted for age, BMI, ethnicity, batch effects, and Bonferroni correction. Associations significant after Bonferroni correction are in bolded text.

*β* Log_2_FC, *FC* fold-change.

^a^For employment duration, effect is for a 6 month increase. For fire-hours, effect is for a 10 h increase. For fire-runs, effect is for a 10 fire increase.

^b^Based on the highest Akaike information criteria (AIC), the best measure of time since most recent fire was selected (continuous, split at median value, split at tertile values, or split at quartile values). Hsa-miR-26a-5p, hsa-miR-548a-3p, hsa-miR-556-3p and hsa-let-7f-5p used time split at the median. Hsa-miR-494-3p, hsa-miR-422a, hsa-miR-92a-3p and hsa-miR-548ad-3p used time split at quantiles. Hsa-miR-525-3p used time split at the median for the model adjusted for structure fire-hours and time split at tertiles for the model adjusted for structure fire-runs.

^c^An absolute FC >1.25 was applied to statistically significant miRNAs associated with length of service presented here.

The results of all other full miRNA analyses are described below. In our univariable models (adjusted for age, ethnicity, BMI, batch effects, and multiple comparisons) for all fires, employment duration, fire-hours and fire-runs were all significantly associated with over 50% of miRNAs in the full array, and time since most recent fire exposure was associated with over 20% of all miRNAs (results not shown). When we considered the full array of miRNAs in multivariable models for all fires, employment duration was associated with four miRNAs (miR-422a, miR-525-3p, miR-548ad-3p and miR-548k) with absolute fold changes >1.25 (Supplementary Table 1). In the multivariable models of all fires, no miRNAs were significantly associated with fire-hours or fire-runs. In our univariable models restricted to structure fires, employment duration, structure fire-hours, and structure fire-hours were each significantly associated with over 50% of all miRNAs in the full array and time since most recent structure fire exposure was associated with over 20% of all miRNAs (results not shown).

## Discussion

The results of this study build on our previous comparison of incumbent and new recruit firefighters which identified nine whole blood miRNAs with at least 50% difference in expression between the two groups [16]. Using these nine miRNAs as a priori markers in our current analysis, we found three (miR-1260a, miR-5010-3p and miR-486-3p) in the multivariable models that were significantly associated with employment duration in the new recruits, all of which were in the same direction of effect as in the previous study, albeit of lesser magnitude. The consistency of these findings between the previous and current study suggests that these miRNAs may serve as biomarkers of cumulative effect in firefighters. In addition, nine novel miRNAs out of the full array panel with longitudinal fold changes of at least 25% were also found to be associated with employment duration in multivariable models restricted to structural fires, as well as four miRNAs with longitudinal fold changes of at least 25% associated with employment duration in multivariable models for all fires, with three of the miRNA present in both all fire and structural fire models. Given the overall pattern of reduction in tumor suppressor miRNAs and increase in oncogenic miRNAs, these findings also provide potential mechanisms linking firefighting to increased cancer risk. In relation to cumulative measures of fireground exposure, in the fully adjusted models restricted to structural fires one a priori miRNA was associated with fire-runs, suggesting a potential dose-response relationship with exposure to products of combustion. Furthermore, the statistically significant association between some miRNAs and most recent fire demonstrates the importance of considering both chronic and acute exposures.

A limited number of previously published studies have also evaluated epigenetic modifications among firefighters. In our two previous studies, we compared incumbent to new recruit firefighters for significant differences in miRNAs and DNA methylation using full array panels of markers [16, 18]. For miRNAs, we identified nine markers with a statistically significant >50% difference in expression in incumbent firefighters compared to new recruits. Results from an enrichment analysis of miRNA clusters also identified associations with stem cells, inflammation pathways, carcinomas, Burkitt’s lymphoma, and melanoma, among others [16]. Our previous study of DNA methylation at cytosine-guanine dinucleotides (CpG) sites among firefighters identified four with a statistically significant >50% difference in methylation in incumbents compared to new recruits [18]. We were also able to demonstrate that genome-wide methylation could predict with accuracy incumbent and new recruit status as well as employment duration among incumbent firefighters. Another study examined DNA methylation among firefighters at four specific genes and observed decreased methylation at the promoter region of one gene, *DUSP22*, in firefighters compared to non-firefighters, correlated with employment duration but not age [17]. The authors also demonstrated through in vitro tests that decreased *DUSP22* promoter methylation was inducible by low-dose benzo[a]pyrene [17], one of a large number of PAHs formed as a combustion byproduct of organic materials. Certain PAHs, including benzo[a]pyrene, are known or probable carcinogens [38]. Based on these associations, the authors concluded that PAHs, a pervasive fire service exposure [4] may contribute to certain cancer risk through epigenetic mechanisms in firefighters due to chronic occupational exposure.

Surrogate fireground measures have been associated with cancer risk among firefighters in a pooled-cohort analysis of three metropolitan career fire departments in the US, including significantly increased lung cancer and mortality risk associated with fire-hours and similar but non-significant associations with fire-runs as well as a marginally significant association between leukemia mortality and fire-runs but not fire-hours [7]. In our multivariable models of a priori miRNAs, structure fire-runs, but not all fire-runs, were significantly associated with decreased expression of miR-486-3p. The direction of this association for structure fire-runs was unexpected as in this same model miR-486-3p increased significantly with employment duration and also non-significantly with most recent fire. This difference in outcomes between structure fire-runs and all fire-runs may be explained by higher exposures of certain combustion byproducts at structure fires compared to vehicle fires [3]. Previous epidemiologic studies of firefighter cancer that were able to assess for dose-response relationships have typically not differentiated between all fires and structure fires [7]. The biological consequences of the negative association of miR-486-3p with structure fires remains to be determined. Similar to some other miRNAs, miR-486-3p has been shown to manipulate several target genes, in this case acting as an oncogene for some cancers (colon, laryngeal squamous cell cancers) and a tumor suppressors for other cancers (oral squamous cell, lung, cervical cancers) [21, 31].

We identified nine novel miRNAs with at least a 25% fold longitudinal change associated with employment duration in our full array analyses restricted to structural fires. Four of the five miRNAs with decreased expression are reported as being tumor suppressors and the four with increased expression are reported as being oncogenic in various cancers, including colorectal, liver, prostate, breast, and bone cancers [35, 36, 39–41]. Of these, previous studies have found that colorectal and prostate cancers are elevated in firefighters [6, 8, 10, 11]. Given that both decreased expression of tumor suppressive and increased expression of oncogenic miRNAs have been associated with increased risk of cancer diagnosis and prognosis [42], our current findings could help explain the excess of certain cancers in firefighters.

In most of our multivariable analyses, we did not observe significant associations between surrogate measures of fireground exposure and dysregulated miRNA expression. Given the relatively small sample size of our current study, we likely lacked sufficient power to detect additional significant associations. However, the majority of firefighter responses are to medical aid calls [43], and other firefighter occupational factors, such as total call volume, stress, shiftwork, and firehouse exposures like diesel exhaust, might help explain dysregulated miRNA expression and should be evaluated in future analyses. Psychological stress can elicit alterations in human whole blood miRNA [44]. Other studies have observed that exposure to diesel exhaust is associated with epigenetic modifications, including dysregulated miRNA [17, 45]. Additionally, while miRNA expression has not been examined for potential associations with shiftwork to our knowledge, aberrant DNA methylation has been observed in long-term night shiftworkers [46]. Finally, we did not include training fire hours in the exposure metrics, as training fires burn different materials (wood, hay and natural gas) than with the exception of wood are present in fires in the community and training fire hours were evenly distributed across the study participants.

The current study findings are consistent with both chronic and acute contributions of firefighter exposures to longitudinal change in whole blood miRNA. Relative to our prior study comparing whole blood miRNA in incumbent firefighters with an average of 14.1 years of occupational exposure compared to new recruits [16], the fold changes over 20–37 months in some of the a priori miRNA markers of the current study were lesser in magnitude. This suggests that changes in at least some whole blood miRNA may reflect cumulative exposure over multiple years, and that larger and longer-term prospective cohort studies are needed to provide additional information on the slope of the dose-response curve over time. Furthermore, while our study found multiple miRNAs with significant associations with time since most recent fire, additional research is also needed to better understand the nature and timing of these relationships. We were able to find a limited number of other published studies on the relation of blood miRNA to acute exposures. A study of steel plant workers examined the effect of particulate matter on miRNA expression using peripheral blood leukocytes (a component of whole blood) and found that after three days of work, expression of two of three candidate miRNAs were significantly different and also correlated with blood oxidative stress or lead exposure [47]. A controlled study of asthmatics exposed to diesel exhaust collected whole blood samples from participants before and six hours after exposure. This study reported that expression of several miRNAs were associated with acute moderate-dose diesel exhaust exposure [45]. Additionally, some changes in miRNA appear to be reversible with cessation of exposure. A small study reported that while miRNA profile of smokers were significantly different than that of non-smokers, after ~1 month the miRNA profile of individuals who independently decided to quit smoking during the study period resembled that of non-smokers [48]. Another study found that out of 34 miRNA dysregulated by smoking in small airway epithelium, 22 returned to normal within three months of cessation of exposure [49]. In vitro studies have found increases in miRNA expression within hours of exposure [50]. These collective findings indicate the need to consider the contribution of both chronic and acute exposures to epigenetic changes.

To our knowledge, this is the first longitudinal analysis of epigenetic changes in firefighters and the first longitudinal assessment of surrogate fireground exposures associated with changes in miRNA expression. We were able to enroll a cohort of new-recruit firefighters with no previous occupational smoke exposure and collect baseline blood samples prior to live-fire training, allowing us to capture a more accurate assessment of miRNA expression prior to any fireground exposures for comparison to samples collected ~2 years later. Collection of fire response history for each study participant allowed us to examine potential dose-response relationships between surrogate fireground exposures and change in miRNA expression as well as evaluate and adjust chronic surrogate measures of fireground exposure (cumulative fire-hours and fire-runs) for potential confounding by acute fireground exposures (days between follow-up blood draw and most recent fire response).

Despite our study’s strengths, there were limitations in regards to both exposure assessment and miRNA measurement. Though we were able to capture administrative information regarding type of fire and our study sample was limited to firefighters within the first two or three years of their career (excluding ranks or responsibilities that require tenure and additional training such as paramedics or engineers), we were unable to account for potential confounding by job assignment (e.g., interior or exterior role at fire). In regards to miRNA measurement, our samples collected at baseline and follow-up were analyzed in four separate batches. To address batch effects, we employed the newer RUV-III correction approach, rather than older methods of batch effect correction such as the ComBat function [51]. Use of methods such as ComBat to remove batch effects in such a situation could lead to biasing (usually deflating) group differences [52]. Finally, the study findings are from one fire department and additional studies in other geographic locations are needed to determine their generalizability.

In conclusion, consistent with previous research, our study provides further evidence that miRNAs may serve as biomarkers of cumulative exposure to firefighters, although the influence of acute exposures and the entirety of firefighter occupational exposures (shiftwork, stress, and other factors in addition to combustion byproducts) requires further investigation. Additionally, we observed significant dose-response relationships between employment duration and dysregulated expression of a priori miRNAs implicated in cancers and cancer pathways. Together, our study provides evidence that alterations of miRNA expression may serve as a mechanism linking firefighter exposures to increased cancer risk.

## Supplementary information

Supplementary Information

## References

[1] Fent KW, Toennis C, Sammons D, Robertson S, Bertke S, Calafat AM (2020). Firefighters’ absorption of PAHs and VOCs during controlled residential fires by job assignment and fire attack tactic. J Expo Sci Environ Epidemiol.

[2] Evans DE, Fent KW (2015). Ultrafine and respirable particle exposure during vehicle fire suppression. Environ Sci Process Impacts..

[3] Bolstad-Johnson DM, Burgess JL, Crutchfield CD, Storment S, Gerkin R, Wilson JR (2000). Characterization of firefighter exposures during fire overhaul. AIHAJ..

[4] Stec AA, Dickens KE, Salden M, Hewitt FE, Watts DP, Houldsworth PE (2018). Occupational exposure to polycyclic aromatic hydrocarbons and elevated cancer incidence in firefighters. Sci Rep..

[5] Burgess JL, Nanson CJ, Bolstad-Johnson DM, Gerkin R, Hysong TA, Lantz RC (2001). Adverse respiratory effects following overhaul in firefighters. J Occup Environ Med..

[6] Daniels RD, Kubale TL, Yiin JH, Dahm MM, Hales TR, Baris D (2014). Mortality and cancer incidence in a pooled cohort of US firefighters from San Francisco, Chicago and Philadelphia (1950–2009). Occup Environ Med..

[7] Daniels RD, Bertke S, Dahm MM, Yiin JH, Kubale TL, Hales TR (2015). Exposure-response relationships for select cancer and non-cancer health outcomes in a cohort of U.S. firefighters from San Francisco, Chicago and Philadelphia (1950–2009). Occup Environ Med..

[8] Jalilian H, Ziaei M, Weiderpass E, Rueegg CS, Khosravi Y, Kjaerheim K (2019). Cancer incidence and mortality among firefighters. Int J Cancer..

[9] Tsai RJ, Luckhaupt SE, Schumacher P, Cress RD, Deapen DM, Calvert GM (2015). Risk of cancer among firefighters in California, 1988–2007. Am J Ind Med..

[10] Lee DJ, Koru-Sengul T, Hernandez MN, Caban-Martinez AJ, McClure LA, Mackinnon JA (2020). Cancer risk among career male and female Florida firefighters: evidence from the Florida Firefighter Cancer Registry (1981–2014). Am J Ind Med..

[11] LeMasters GK, Genaidy AM, Succop P, Deddens J, Sobeih T, Barriera-Viruet H (2006). Cancer risk among firefighters: a review and meta-analysis of 32 studies. J Occup Environ Med..

[12] IARC. Painting, firefighting, and shiftwork: Lyon (FR); 2010. http://monographs.iarc.fr/ENG/Monographs/vol98/mono98.pdf. PMC4781497 21381544

[13] Biswas S, Rao CM (2017). Epigenetics in cancer: fundamentals and beyond. Pharm Ther..

[14] Humphries B, Wang Z, Yang C (2019). MicroRNA regulation of epigenetic modifiers in breast cancer. Cancers..

[15] Azmi AS, Beck FWJ, Bao B, Mohammad RM, Sarkar FH (2011). Aberrant epigenetic grooming of miRNAs in pancreatic cancer: a systems biology perspective. Epigenomics..

[16] Jeong KS, Zhou J, Griffin SC, Jacobs ET, Dearmon-Moore D, Zhai J (2018). MicroRNA changes in firefighters. J Occup Environ Med..

[17] Ouyang B, Baxter CS, Lam H-M, Yeramaneni S, Levin L, Haynes E (2012). Hypomethylation of dual specificity phosphatase 22 promoter correlates with duration of service in firefighters and is inducible by low-dose benzo[a]pyrene. J Occup Environ Med..

[18] Zhou J, Jenkins TG, Jung AM, Jeong KS, Zhai J, Jacobs ET (2019). DNA methylation among firefighters. PLoS One..

[19] Kwon JJ, Factora TD, Dey S, Kota J (2018). A systematic review of miR-29 in cancer. Mol Ther Oncolytics..

[20] Esquela-Kerscher A, Slack FJ (2006). Oncomirs - microRNAs with a role in cancer. Nat Rev Cancer.

[21] ElKhouly AM, Youness RA, Gad MZ (2020). MicroRNA-486-5p and microRNA-486-3p: multifaceted pleiotropic mediators in oncological and non-oncological conditions. Noncoding RNA Res..

[22] Wahid F, Shehzad A, Khan T, Kim YY (2010). MicroRNAs: synthesis, mechanism, function, and recent clinical trials. Biochim Biophys Acta..

[23] Griffiths-Jones S, Grocock RJ, van Dongen S, Bateman A, Enright AJ (2006). miRBase: microRNA sequences, targets and gene nomenclature. Nucleic Acids Res.

[24] Dennis L, Rhodes M, Maclean K. Targeted miRNA discovery and validation using the nCounter® platform (white paper). Seattle, WA: NanoString Technologies, Inc; 2015.

[25] Molania R, Gagnon-Bartsch JA, Dobrovic A, Speed TP (2019). A new normalization for nanostring nCounter gene expression data. Nucleic Acids Res..

[26] Ritchie ME, Phipson B, Wu D, Hu Y, Law CW, Shi W (2015). limma powers differential expression analyses for RNA-sequencing and microarray studies. Nucleic Acids Res.

[27] Smyth GK (2004). Linear models and empirical bayes methods for assessing differential expression in microarray experiments. Stat Appl Genet Mol Biol.

[28] Sedgwick P (2014). Multiple hypothesis testing and Bonferroni’s correction. BMJ: Br Med J.

[29] Dahm MM, Bertke S, Allee S, Daniels RD (2015). Creation of a retrospective job-exposure matrix using surrogate measures of exposure for a cohort of US career firefighters from San Francisco, Chicago and Philadelphia. Occup Environ Med..

[30] Posada D, Buckley TR (2004). Model selection and model averaging in phylogenetics: advantages of akaike information criterion and bayesian approaches over likelihood ratio tests. Syst Biol..

[31] Mosakhani N, Sarhadi VK, Borze I, Karjalainen-Lindsberg ML, Sundstrom J, Ristamaki R (2012). MicroRNA profiling differentiates colorectal cancer according to KRAS status. Genes Chromosomes Cancer..

[32] Lin K, Xu T, He B-S, Pan Y-Q, Sun H-L, Peng H-X (2016). MicroRNA expression profiles predict progression and clinical outcome in lung adenocarcinoma. Onco Targets Ther..

[33] Tsai H-P, Huang S-F, Li C-F, Chien H-T, Chen S-C (2018). Differential microRNA expression in breast cancer with different onset age. PLoS One..

[34] Zheng G, Du L, Yang X, Zhang X, Wang L, Yang Y (2014). Serum microRNA panel as biomarkers for early diagnosis of colorectal adenocarcinoma. Br J Cancer.

[35] Augello C, Vaira V, Caruso L, Destro A, Maggioni M, Park YN (2012). MicroRNA profiling of hepatocarcinogenesis identifies C19MC cluster as a novel prognostic biomarker in hepatocellular carcinoma. Liver Int..

[36] Sugita B, Gill M, Mahajan A, Duttargi A, Kirolikar S, Almeida R (2016). Differentially expressed miRNAs in triple negative breast cancer between African-American and non-Hispanic white women. Oncotarget..

[37] Zhang W, Hong R, Li L, Wang Y, Du P, Ou Y (2018). The chromosome 11q13.3 amplification associated lymph node metastasis is driven by miR-548k through modulating tumor microenvironment. Mol Cancer..

[38] IARC. Benzo[a]pyrene. In: Chemical agents and related occupations: Lyon (FR)IARC monographs on the evaluation of carcinogenic risks to humans. 2012. https://monographs.iarc.fr/wp-content/uploads/2018/06/mono100F-14.pdf. PMC4781612 23189753

[39] Cai B, Peng JH. Increased expression of miR-494 in serum of patients with prostate cancer and its potential diagnostic value. Clin Lab. 2019;65. 10.7754/Clin.Lab.2019.190422. 10.7754/Clin.Lab.2019.190422 31414754

[40] Slattery ML, Mullany LE, Sakoda LC, Wolff RK, Samowitz WS, Herrick JS (2018). Dysregulated genes and miRNAs in the apoptosis pathway in colorectal cancer patients. Apoptosis..

[41] Xie L, Liao Y, Shen L, Hu F, Yu S, Zhou Y (2017). Identification of the miRNA-mRNA regulatory network of small cell osteosarcoma based on RNA-seq. Oncotarget..

[42] Drebber U, Lay M, Wedemeyer I, Vallbohmer D, Bollschweiler E, Brabender J (2011). Altered levels of the onco-microRNA 21 and the tumor-supressor microRNAs 143 and 145 in advanced rectal cancer indicate successful neoadjuvant chemoradiotherapy. Int J Oncol..

[43] NFPA. NFPA statistics - fire department calls. 2019. https://www.nfpa.org/News-and-Research/Data-research-and-tools/Emergency-Responders/Fire-department-calls.

[44] Gidron Y, De Zwaan M, Quint K, Ocker M (2010). Influence of stress and health-behaviour on miRNA expression. Mol Med Rep..

[45] Yamamoto M, Singh A, Sava F, Pui M, Tebbutt SJ, Carlsten C (2013). MicroRNA expression in response to controlled exposure to diesel exhaust: attenuation by the antioxidant N-acetylcysteine in a randomized crossover study. Environ Health Perspect.

[46] Liu R, Jacobs DI, Hansen J, Fu A, Stevens RG, Zhu Y (2015). Aberrant methylation of miR-34b is associated with long-term shiftwork: a potential mechanism for increased breast cancer susceptibility. Cancer causes Control..

[47] Bollati V, Marinelli B, Apostoli P, Bonzini M, Nordio F, Hoxha M (2010). Exposure to metal-rich particulate matter modifies the expression of candidate microRNAs in peripheral blood leukocytes. Environ Health Perspect.

[48] Takahashi K, Yokota S-i, Tatsumi N, Fukami T, Yokoi T, Nakajima M (2013). Cigarette smoking substantially alters plasma microRNA profiles in healthy subjects. Toxicol Appl Pharmacol..

[49] Wang G, Wang R, Strulovici-Barel Y, Salit J, Staudt MR, Ahmed J (2015). Persistence of smoking-induced dysregulation of miRNA expression in the small airway epithelium despite smoking cessation. PLoS One..

[50] Rager JE, Smeester L, Jaspers I, Sexton KG, Fry RC (2011). Epigenetic changes induced by air toxics: formaldehyde exposure alters miRNA expression profiles in human lung cells. Environ Health Perspect..

[51] Leek JT, Johnson WE, Parker HS, Jaffe AE, Storey JD (2012). The sva package for removing batch effects and other unwanted variation in high-throughput experiments. Bioinforma..

[52] Nygaard V, Rødland EA, Hovig E. Methods that remove batch effects while retaining group differences may lead to exaggerated confidence in downstream analyses. Biostatistics. 2016;17:29–39. 10.1093/biostatistics/kxv027. 10.1093/biostatistics/kxv027 PMC4679072 26272994

[53] Tan Y, Ge G, Pan T, Wen D, Chen L, Yu X (2014). A serum microRNA panel as potential biomarkers for hepatocellular carcinoma related with hepatitis B virus. PLoS ONE.

[54] Ghanbari R, Mosakhani N, Sarhadi VK, Armengol G, Nouraee N, Mohammadkhani A (2016). Simultaneous Underexpression of let-7a-5p and let-7f-5p microRNAs in Plasma and Stool Samples from Early Stage Colorectal Carcinoma. Biomark Cancer.

[55] Nguyen HCN, Xie W, Yang M, Hsieh C-L, Drouin S, Lee G-SM (2013). Expression differences of circulating microRNAs in metastatic castration resistant prostate cancer and low-risk, localized prostate cancer. The Prostate.

