# Supplementary material for: Longitudinal evaluation of whole blood miRNA expression in firefighters
Source: J Expo Sci Environ Epidemiol. Author manuscript; Available in PMC 2021 Oct 12. (PMC8445815; doi:10.1038/s41370-021-00306-8)
Supplement: Supplementary Information for: Longitudinal evaluation of whole blood miRNA expression in firefighters [file NIHMS1739757-supplement-Supplementary_Information_for__Longitudinal_evaluation_of_whole_blood_miRNA_expression_in_firefighters.docx]

**Supplementary Information for: Longitudinal evaluation of whole blood miRNA expression in firefighters**

Alesia M Jung^1^, Jin Zhou^1^, Shawn C Beitel^2^, Sally R Littau^2^, John J Gulotta^3^, Darin D Wallentine^3^, Jefferey L Burgess^2^

**Affiliations:**

1. Department of Epidemiology and Biostatistics, Mel and Enid Zuckerman College of Public Health, University of Arizona, Tucson, Arizona

2. Department of Community, Environment and Policy, Mel and Enid Zuckerman College of Public Health, University of Arizona, Tucson, Arizona

3. Tucson Fire Department, Tucson, Arizona

**Address correspondence to**: Jefferey L. Burgess, MD, MS, MPH, Mel and Enid Zuckerman College of Public Health, University of Arizona, 1295 N Martin Ave, Tucson, AZ 85724; Fax number (520) 626-6093; Telephone number (520) 626-4918; Email address [jburgess@email.arizona.edu](mailto:jburgess@email.arizona.edu)

**Included in this file:**

**Supplementary Table 1**

**Table of Contents**

**Supplemental Table 1.** Parameter estimates of statistically significant miRNAs from full miRNA expression panel associated with employment duration and adjusted for fire-hours or fire-runs and time since most recent fire……………………………………………………… 3

| Supplemental Table 1. Parameter estimates of statistically significant miRNAs from full miRNA expression panel associated with employment duration and adjusted for fire-hours or fire-runs and time since most recent fire^a, b, c^ | | | | | | | | | | | | | | |
| --- | --- | --- | --- | --- | --- | --- | --- | --- | --- | --- | --- | --- | --- | --- |
|  | **Model with fire-hours** | | | | | | | **Model with fire-runs** | | | | | | |
| miRNA | Employment duration | | Fire-hours | | Most recent fire | | R^2 | Employment duration | | Fire-runs | | Most recent fire | | R^2 |
|  | β | p-value | β | p-value | β | p-value |  | β | p-value | β | p-value | β | p-value |  |
| hsa-miR-422a | **-0.341** | **<0.001** | 0.169 | 0.639 | 0.169 | 0.639 | 0.594 | - | - | - | - | - | - | - |
| hsa-miR-525-3p | **0.471** | **<0.001** | 0.190 | 0.003 | **-1.906** | **<0.001** | 0.560 | - | - | - | - | - | - | - |
| hsa-miR-548ad-3p | **0.527** | **<0.001** | 0.238 | 0.240 | -2.051 | <0.001 | 0.452 | - | - | - | - | - | - | - |
| hsa-miR-548k | - | - | - | - | - | - | - | **0.513** | **<0.001** | -0.136 | 0.394 | -2.015 | <0.001 | 0.221 |
| β = Log_2_FC; FC= fold-change;  Models also adjusted for age, BMI, ethnicity, batch effects, and Bonferroni correction. Associations significant after Bonferroni correction are in bolded text.  ^a^ For employment duration, effect is for a 6 month increase. For fire-hours, effect is for a 10 hour increase. For fire-runs, effect is for a 10 fire increase.  ^b^ Based on the highest Akaike information criteria (AIC), the best measure of time since most recent fire was selected (continuous, split at median value, split at tertile values, or split at quartile values). Hsa-miR-422a used time split at the median. hsa-miR-525-3p, hsa-miR-548ad-3p, and hsa-miR-548k used time split at quantiles.  ^c^ An absolute FC >1.25 was applied to statistically significant miRNAs associated with length of service presented here. | | | | | | | | | | | | | | |
